# Editorial: Integrative Approaches to the Molecular Physiology of Inflammation

**DOI:** 10.3389/fphys.2018.01825

**Published:** 2018-12-18

**Authors:** Enrique Hernández-Lemus, Maria Elena Soto, Carlos Rosales

**Affiliations:** ^1^Computational Genomics, Instituto Nacional de Medicina Genómica, Mexico City, Mexico; ^2^Departamento de Inmunología, Instituto Nacional de Cardiología Ignacio Chávez, Mexico City, Mexico; ^3^Departamento de Inmunología, Instituto de Investigaciones Biomédicas, Universidad Nacional Autónoma de México, Mexico City, Mexico

**Keywords:** inflammation, molecular physiology, integrative biology, immunity, pathophyisology

Inflammation is the generic name given to a number of complex biological processes related to the organismal response to a disparate set of stimuli (most of them harmful or pathogenic), either intrinsic (DNA damage, metabolic deregulation, etc.) or extrinsic (pathogens, irritants, etc.) in nature. Such processes are commonly related to a protective reaction to disease related events that involve immune response, vascularization, and cellular signaling among many other features. Recent years have witnessed an increased interest in the study of inflammation, since it was discovered that inflammatory processes are associated with a growing number of pathologies, many of which had not been previously classified as “inflammatory.”

Complex chronic diseases such as cancer, diabetes, or even Alzheimer's or Parkinson's have recently been discovered to be strongly associated with inflammatory responses. Other maladies such as cardiovascular, rheumatic, and autoimmune diseases have been traditionally studied from the standpoint of inflammation. Processes such as the ones leading to aging and fragility or even hormone dysfunctions are also starting to be related to inflammatory responses.

The various stages and processes of inflammation can be related to genetic changes, environmental and molecular patterns associated with damage (DAMPs) or molecular patterns associated with microbes (MAMPs) or molecular patterns associated with pathogens (PAMPs). These molecular patterns, when recognized by different cells, can induce multiple vascular and cellular responses that lead to the clinical diversity of inflammatory conditions related to pathogenesis.

For the reasons mentioned above, an important goal in contemporary biomedical science is the integrated study of the physiology of inflammation and the molecular pathways associated with it. The aim of this Research Topic is hence to gather contributions from the many different fields and approaches to the physiology and the molecular origins of inflammation; particularly those that may be involved in the development and evolution of diseased phenotypes. By presenting them together we want to cooperate to unveil the commonalities and differences that so many of these phenomena have, particularly in relation to their molecular origins as well as to any issues that may enlighten prognostics, diagnostics, and therapeutic decisions.

We believe that this Research Topic on Integrative Approaches to the Molecular Physiology of Inflammation is a good selection of the wide scope and multiple (seemingly disparate but often convergent) views to approach the molecular origins of complex inflammatory phenotypes. It consists on 24 articles: 17 original research contributions and 7 review articles (5 reviews, 1 systematic review, and 1 minireview). Both, the research papers and the reviews provide varied and insightful approaches to the different facets of inflammation with approaches ranging from general inflammation and signaling depictions deeply rooted on functional biology and physiology, to computational systems biology analyses, translational medicine, and pharmacological explorations. Model systems are also quite diverse: human subjects, mice and other mammal models, cell cultures and *in silico*, complex networks and database studies.

These systems allowed the analysis of questions that include: dissecting the inflammatory components of complex disease phenotypes, such is the case of the review by Homme et al. on diabetic retinopathy and its relationship with pyroptosis as well as downstream products of inflammation; and of the research paper by Sheffield et al. where they present their findings on how deregulation of inflammation leads to changes in cartilage homeostasis with strong consequences for Bardet-Biedl cilliopathy.

Inflammatory processes are often intertwined with abnormal metabolic regulation, such is the case of inflammation induced changes in the arachidonic acid metabolism leading to disruption of the aortic function of patients with Marfan syndrome. In the aortic tissue of these patients, it has been shown that the inflammatory process through an imbalance in the synthesis of prostaglandins is a consequence of a differential protein expression of the isoforms of cyclooxygenases (COXs) COX1 and COX2. This imbalance ultimately contributes to distorted vascular smooth muscle contraction and relaxation. Also, the overexpression of COX2 induces metalloproteinases whose activation directs the degradation of the extracellular matrix. Together, these effects lead to a significant increase in the aortic aneurysm of patients with Marfan syndrome as presented in the article by Soto et al.

Related changes in metabolism, driven by inflammasome activity and oxidative stress may even challenge therapeutic interventions in diabetic patients with cardiovascular complications as is thoroughly reviewed by the Sharma et al. The multiple roles of neutrophils in inflammation are thoroughly explored in the review paper by Rosales. Systemic chronic low-grade inflammatory processes are also associated with protein metabolism and anabolic sensitivity in age-related sarcopenia as it is discussed by Dalle et al.

A functional approach to study differential signaling networks related to autocrine processes in hepatic stellate cells and hepatocytes under stress is presented by Vodovotz et al. The authors were able to differentiate between networks associated with intracellular information processing (“thinking”) and networks devoted to extracellular information transfer (“talking”), whose interplay results determinant for the differences in autocrine response in both cell types. Also in connection with systemic signaling regulation, the research paper by Bösl et al. provide a nice example of cooperative signaling by two TLRs, enhancing the regulatory processes in a way no single receptor cascades may actually achieve. The way in which system-wide molecular processes affect tissues and physiological behaviors is well exemplified by the research article contributed by Jarkovska et al. The authors identified cellular and molecular mechanisms behind the loss of cardiac tone by myocardial depression, a condition associated with septic shock. The associated molecular mechanisms constitute a novel tool to unveil potential therapeutic targets useful for the prevention and treatment of sepsis-induced myocardial dysfunction.

The relation between metabolic processes in the muscular tissue and inflammation is further discussed in the research paper by Kim et al. on IL-1β production in vascular smooth muscle cells and in the work by Miao et al. on the attenuation of cancer cachexia by crosstalk induction by inflammation products.

The molecular origins of inflammatory processes affecting the architecture and function of the endothelium have also been covered in this special issue: On the one hand, the work of Wang and Lo identifies the action of the basic fibroblast growth factor on protecting the laminar shear flow medium of the arteries from the action of TNF-alpha induced endothelial dysfunction. On the other hand, the same group identified microRNA-mediated changes in glucose metabolism leading to endothelial inflammation, as it can be found in the manuscript by Lo et al. Also discussing the role of non-coding RNAs in inflammation is the systematic review article by Gao et al., there they present an up to date review as well as a meta-analysis of the prognostic applications of lncRNA GAS5 in several carcinomas.

Neurological disorders have also been linked to non-resolved inflammation scenarios. In this regard, an integrative computational approach by the group of Ravichandran et al. shows how the analysis of molecular networks involved in inflammation led to the discovery of specific sites linked to Alzheimer's disease. Mathematical and computational models are increasingly providing insight, not only in the pathophysiology of inflammation and its influence on disease but are also helpful in the development of systematic therapeutics. Particularly useful are approaches that allow drug repurposing, since these provide a significantly faster and easier transition from research findings to patients' treatment. The work by de Anda-Jáuregui et al. provides a powerful example of high-throughput computational analysis of massive experimental databases combined with a network approach to the repositioning of anti-inflammatory drugs.

The development of novel therapeutic strategies and pharmacological approaches for the modulation of inflammatory processes is deeply covered in this research topic. Gao et al. contributed with a review that covers some of the therapeutic uses of the TLR signaling pathway in the development of anti-inflammatory therapeutics based on molecular approaches going up to nanomedicine. There are also a number of research papers dealing with pharmacological and therapeutical developments to treat inflammation. This is the case of the study of the effects of the small molecule LASSBio-897 to treat silicosis as discussed by Carvalho et al.

The use of plant-derived pharmaceuticals to treat inflammation is covered by the study on the effects of the extract of the *Amomum villosum* ginger (rich in bornyl acetate) to treat intestinal mucositis as presented in the contribution by Zhang et al. Another natural product with important anti-inflammatory effects is carnosic acid, a benezenediol compound with anti-oxidant properties that can be extracted from both rosemary and sage. In their contribution, Zhang et al. showed that by an epigenomic mechanism of miR-29b-3p-mediated inhibition of HMGB1/TLR4 carnosic acid is able to alleviate liver fibrosis in an animal model. The use of isoproterenol to modify aortic vasoreactivity and VCAM-1 modulation was investigated in an animal model and reported by Nieto-Lima et al. They found important age and gender associations between drug dose and response to the treatment. The differences found are attributed to differential beta-adrenergic stimulation. Authors discuss how then it is relevant to consider age and gender variability in the design of animal models, as it is customarily made in the clinical settings. In an additional animal model investigation, Trojan et al. developed an in-depth network study of chemokine interactions of anti-depressants that unveil the role of such drugs on the modulation of chemokine-chemokine interactions. The mini review by Cavalcante-Silva et al. on the modulatory properties of ouabain highlights a broader applicability of this cardiotonic steroid that includes regulatory actions of inflammatory events such as cell migration, vascular permeability, and cytokine production. Therapeutics is highly benefitted from translational research studies. In the case of nonalcoholic steatohepatitis, the work by Morrison et al. on gene profiling to unveil key modulators of inflammation in a mouse model of this disease results enlightening by showing that the effects of master regulator molecules and specific inflammation pathways are similar between the human-based and animal models.

Together, these articles provide a sample of the multiple and complex roles of inflammation and its involvement in infections, immunity, and multiple pathological conditions. They also provide guidance for novel therapeutic approaches and future research on the physiology of inflammation.

## Author Contributions

EH-L, MS, and CR planned and edited this special topic. EH-L, MS, and CR wrote this editorial.

### Conflict of Interest Statement

The authors declare that the research was conducted in the absence of any commercial or financial relationships that could be construed as a potential conflict of interest.

